# Oral Health Care for Institutionalized Elderly in Romania: Needs, Barriers, and Legislative Gaps

**DOI:** 10.3390/dj13110527

**Published:** 2025-11-10

**Authors:** Raluca Mioara Cosoroabă, Şerban Talpoş, Ştefania Dinu, Sergio Liga, Alina Doina Tănase

**Affiliations:** 1 Faculty of Dental Medicine, “Victor Babes” University of Medicine and Pharmacy Timisoara, Revolutiei Ave. 1989, No. 9, 300580 Timisoara, Romania; cosoroaba.raluca@umft.ro (R.M.C.); tanase.alina@umft.ro (A.D.T.); 2 Discipline of Oral and Maxillo-Facial Surgery, Faculty of Dental Medicine, “Victor Babes” University of Medicine and Pharmacy Timisoara, 300041 Timisoara, Romania; talpos.serban@umft.ro; 3 Department of Pediatric Dentistry, Faculty of Dental Medicine, “Victor Babes” University of Medicine and Pharmacy Timisoara, Eftimie Murgu Square 2, 300041 Timisoara, Romania; 4 University Clinic of Toxicology, Drug Industry, Management and Legislation, Faculty of Pharmacy, “Victor Babes” University of Medicine and Pharmacy Timisoara, Eftimie Murgu Square No. 2, 300041 Timisoara, Romania; sergio.liga96@gmail.com

**Keywords:** oral health, elderly care, edentulism, dental access, public health policy, legislative frameworks

## Abstract

**Background**: Elderly individuals living in Romanian long-term care facilities encounter substantial challenges in accessing oral healthcare, despite the high prevalence of dental disease and prosthetic needs. This study aimed to evaluate the oral health status, treatment needs, and access barriers among elderly residents in a long-term care facility in Timișoara, Romania, while also analyzing the current legislative framework and public funding mechanisms for geriatric dental care. **Methods**: A cross-sectional clinical and interview-based study was conducted among 70 residents aged 65–89 years from a residential center. Data collection included intraoral examinations, caregiver-assisted surveys, and individual interviews. Outcomes included oral health status (edentulism, caries, prosthetic use), service utilization, perceived barriers, and quality of life impact. The results were compared to existing literature and analyzed within the framework of Romanian and European healthcare legislation. **Results**: Total edentulism was found in 66.6% of participants, with only 28% having functional dentures. Caries and periodontal disease were prevalent, and 84% of residents lacked financial resources for dental care. Only 17% had accessed any dental services in the past year. Structural limitations, including the absence of mobile dental services and the lack of institutional protocols, further restricted access. Romanian Law no. 17/2000 guarantees healthcare in residential settings, but oral health is not explicitly included. **Conclusions**: The study highlights a critical gap in the provision of oral healthcare to institutionalized elderly in Romania. Neglect compromises nutrition, autonomy, and psychosocial well-being, underscoring the urgent need for legislative clarity, dedicated funding, mobile dental services, and integration into geriatric care.

## 1. Introduction

The phenomenon of global aging has a significant impact on health systems and social structures worldwide. By 2050, the proportion of the world’s population aged 60 and over is expected to double, reaching approximately 2 billion individuals. This demographic shift brings a surge in chronic health conditions, with oral health problems being particularly prevalent among older adults [1,2]. Oral health issues in the elderly can range from periodontal disease to tooth loss, often exacerbated by the co-morbidity of other health conditions like dementia or diabetes. Poor oral health is not only a distinct medical issue but is also intricately linked to overall health outcomes, influencing nutritional status, social interactions, and quality of life [2,3].

In Romania, the situation of institutionalized elderly individuals presents a stark representation of these global trends. Access to comprehensive oral health services is severely limited in Romanian care homes, where many elderly patients face economic and bureaucratic barriers that impede necessary dental care [3,4]. The foremost challenges in these institutions include insufficient funding, inadequate staffing, and a general lack of prioritization of oral health services within the healthcare system, as these services often fall under secondary instead of primary health care [4,5]. Furthermore, systemic issues prevail due to the burgeoning demand from an aging population that outpaces the existing healthcare infrastructure, illustrating a critical gap between need and provision.

Common oral issues encountered by elderly individuals include periodontal disease, dental caries, and tooth loss, which are often exacerbated by inadequate access to preventive and therapeutic dental services [6]. Research indicates that elderly residents in long-term care settings often have unmet dental needs, with accessibility barriers contributing to their failure to seek necessary dental care until faced with acute problems [7]. These situations not only diminish the quality of life for elderly patients but can also lead to health complications, as oral health is closely linked to overall health and well-being [8].

In Romania, many elderly patients residing in care homes struggle to access dental services, often due to logistical issues such as transportation and lack of resources within the institutions themselves [9]. Despite legislative frameworks in place, such as the Social Security Law, which allows for the provision of social-medical services, including dental care, the implementation remains inadequate [10].

The Romanian public insurance system, known as the National Health Insurance House (CNAS), significantly limits access to dental care. Coverage is generally restricted to specific treatments and only partially reimburses costs [11,12]. The limitations of the Romanian public insurance system are pronounced, as it covers approximately 60% of oral health services, a statistic consistent with countries classified as low-to-middle income [13]. Often, elderly patients are required to pay out-of-pocket for dental care, which can be financially burdensome and further exacerbate inequalities in health access [10,13]. Consequently, those in care homes may experience a deterioration in their oral health as they cannot afford necessary treatments, resulting in a cycle of neglect and decline in overall health [8,14].

A study by Hage et al. highlighted that the available coverage encompasses only basic dental treatments, primarily focusing on extractions and minimal prosthetic interventions, leaving many necessary procedures uncovered [11]. The existing legislative framework does outline provisions for dental services within social-medical systems; however, implementation has been notably poor, leading to a disconnect between legal entitlements and real-world access [4]. Despite ongoing transformations within the healthcare system, there remains a significant research gap regarding integrated studies exploring the nexus of oral health, economic barriers, and the legislative landscape in Romania. While isolated studies are addressing oral health among the elderly, comprehensive analyses that incorporate sociomedical dimensions, particularly in institutional settings, are conspicuously lacking [3,15].

Thus, the aim of this study was to evaluate the oral health status, treatment needs, and access barriers among elderly residents in long-term care facilities in Romania, while also analyzing the current legislative framework and public funding mechanisms related to geriatric dental care. By addressing these dimensions, the study seeks to contribute evidence-based insights into oral health disparities and to support the development of effective policies and interventions for improving dental care accessibility among this vulnerable population.

## 2. Materials and Methods

### 2.1. Study Design and Setting

This cross-sectional descriptive study was conducted between January and June 2024 at a public residential care center in Timișoara, Romania. The institution houses elderly individuals who are either fully or partially dependent and lack adequate family support.

### 2.2. Selection of Population and Sample

The total institutionalized population consisted of 84 residents. Based on the inclusion and exclusion criteria (Table 1) and after obtaining informed consent, 70 elderly residents were included in the study.

### 2.3. Data Collection Procedures

Oral examination was performed by a licensed dentist using standard dental instruments under ambient light. The following clinical variables were recorded: number of natural teeth, presence of edentulism, dental caries, prosthetic status, periodontal indicators, and oral mucosal lesions. General health data were extracted from patients’ medical records and supplemented by a brief clinical check-up conducted by a physician.

Sociodemographic and perception-related data were collected using a structured 18-item questionnaire, administered with caregiver assistance when necessary. The questionnaire was adapted from previously validated oral health-related quality of life questionnaires and pre-tested on a small group of institutionalized elderly individuals (n = 20) to ensure clarity and reliability prior to data collection. The questionnaire covered:

- Self-perceived oral health and quality of life;
- Previous dental care access;
- Awareness of state-funded services;
- Willingness to accept mobile dental units.

## 3. Results

### 3.1. Demographic Profile of Participants

**Figure 1:**
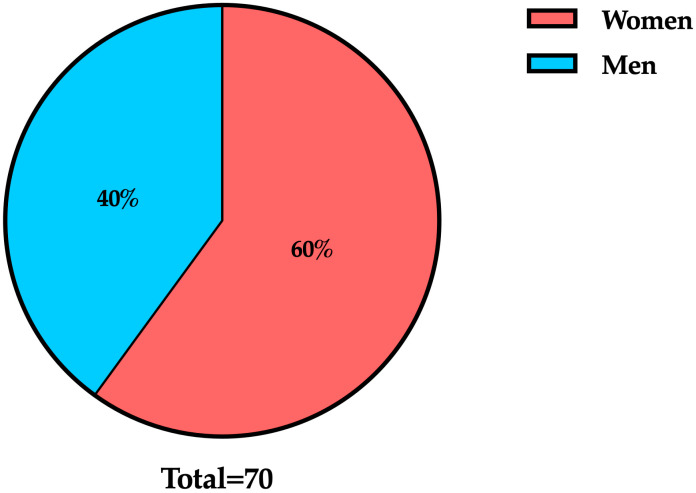
Gender distribution of study participants (n = 70).

The study group consisted of 70 institutionalized elderly individuals residing in a state-funded residential care facility in Timișoara. Participants were aged between 65 and 89 years, with a mean age of 73.8 years (±5.6), reflecting a moderately aged population. The gender distribution was skewed towards females, with 42 women (60%) and 28 men (40%), a pattern that is consistent with national demographic trends, where female longevity contributes to a higher prevalence of women in long-term care settings (Figure 1).

**Figure 2:**
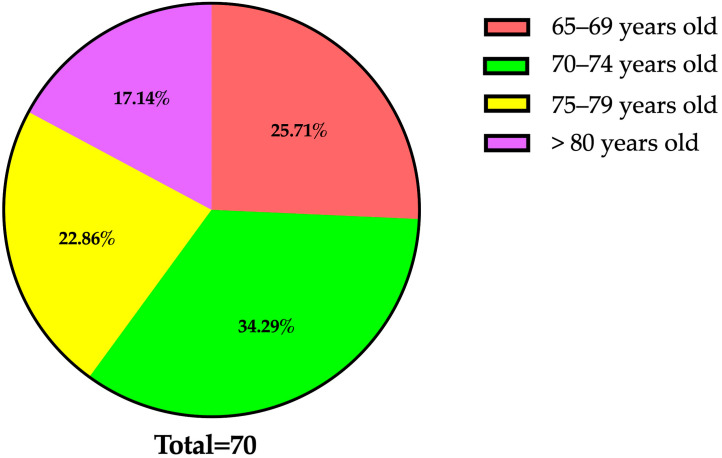
Age distribution of study participants (n = 70; mean age: 73.8 ± 5.6 years).

In terms of age structure (Figure 2), most residents (34.3%) were aged between 70 and 74 years (n = 24), followed by those aged 65 to 69 years (25.7%; n = 18), 75 to 79 years (22.9%; n = 16), and those aged over 80 years (17.1%; n = 12). This age distribution underscores the progressive accumulation of frailty and chronic conditions that often necessitate institutionalization in later life.

Patients were categorized according to their primary chronic conditions, as documented in their medical records. The distribution of the most prevalent comorbidities among long-term care residents is presented in Table 2.

All participants were retired, and over two-thirds (72.8%) reported monthly incomes below 1400 RON, placing them near or under the national poverty threshold. A small number of residents (approximately 18%) reported having no personal income, relying entirely on institutional support for their basic needs. These socioeconomic conditions are highly relevant, as they influence access to healthcare, including dental services, and shape patients’ capacity to engage with preventive or restorative oral health interventions.

### 3.2. Oral Health Status

The clinical oral examination conducted among the 70 participants revealed a substantial burden of untreated dental pathology and prosthetic needs (Table 3).

As summarized in Table 3, total edentulism emerged as the most prevalent oral health condition among the examined residents. This finding was more pronounced among female participants, reflecting both their longer life expectancy and historically limited access to restorative dental care, particularly among older rural-born cohorts. Partial edentulism typically involves posterior tooth loss with significant wear of the remaining anterior teeth. Only a small proportion of residents retained a functionally adequate dentition, highlighting the extent of masticatory impairment in this population.

Regarding prosthetic rehabilitation, 42.9% of participants were completely edentulous and did not possess any form of dentures. An additional 28.6% wore prosthetic devices that were damaged, unstable, or poorly adapted to their current oral anatomy. Only 28.6% had functional complete dentures that ensured satisfactory mastication and speech. Most existing prostheses were reported to be more than 10 years old, showing evident signs of wear and discoloration. Replacement was rare, primarily due to financial limitations and restricted access to dental care services.

In addition to tooth loss, active carious lesions were detected in 38.6% of participants (n = 27). Root caries, often associated with gingival recession and xerostomia, were particularly common among patients with diabetes and cardiovascular conditions.

Periodontal disease was also highly prevalent, with clinical signs of moderate to severe inflammation and attachment loss observed in 44.7% (n = 31) of residents. Of these, 19 patients exhibited mobility in multiple teeth and pocket depths exceeding 6 mm, indicating advanced periodontitis.

Soft tissue abnormalities were found in 13 participants (18.2%), including denture stomatitis, angular cheilitis, and traumatic ulcers. These lesions were often associated with poorly fitting prostheses and inadequate oral hygiene. While no malignant lesions were detected during the examination, the presence of chronic mucosal irritations in vulnerable individuals reinforces the need for routine screening and professional dental oversight.

Overall, these findings highlight the severity of unmet oral health needs in this long-term care resident cohort, underscoring the long-term consequences of limited access to preventive and restorative dental services.

### 3.3. Access and Utilization of Dental Services

The data collected revealed a markedly low utilization of dental services among the study participants over the previous 12 months. Out of the 70 residents included in the analysis, only 12 individuals (16.9%) had accessed any form of dental care within the last year. Most of these visits were prompted by acute conditions, primarily dental pain or the need for tooth extractions, rather than preventive check-ups or prosthetic rehabilitation.

Routine dental consultations were virtually absent, with no participants reporting scheduled prophylactic visits or periodontal maintenance. This lack of engagement with preventive care services highlights a systemic gap in the integration of oral health within the general healthcare protocols for institutionalized elders.

Among the 12 residents who accessed dental services, 10 required emergency interventions (e.g., primarily extractions) due to advanced caries or periapical infections. Only two individuals sought care for denture repair or adjustment, despite the widespread presence of damaged or poorly fitting prostheses in the overall sample.

Access to care was largely dependent on external arrangements: 87% of dental visits (n = 10) required transportation to off-site clinics, typically coordinated by caregivers or family members, resources that were frequently unavailable. Within the institutional complex, only one of the four care units had ever received a mobile dental service, and that visit had occurred more than a year before data collection. None of the facilities had an in-house dental unit or formal partnerships with public or private providers, underscoring the absence of a structured care pathway for residents.

Several participants mentioned having postponed or avoided care due to the complexity of organizing off-site visits, the discomfort associated with transportation, and the perceived indifference of the system to their oral health concerns. These findings align with prior research indicating that institutionalized elderly individuals are among the most underserved groups in terms of access to oral healthcare, despite being at significantly elevated risk for oral disease and its systemic consequences.

Taken together, the observed underutilization of dental services is not reflective of a lack of need, but rather of a network of structural, financial, and organizational barriers that limit the capacity of long-term care individuals to seek and obtain timely oral care.

### 3.4. Perceived Barriers to Oral Health Care

Participants reported multiple, interrelated barriers that severely restricted their access to dental care. Based on structured interviews and caregiver-assisted questionnaires, these obstacles were classified into four main categories: economic, logistical, administrative, and psychological (Table 4).

The most frequently reported barrier to dental care was financial inaccessibility. A large majority of participants indicated that they were unable to afford even basic dental treatments, such as extractions or prosthetic rehabilitation. The average cost of complete acrylic dentures (1600–2000 RON) far exceeded their financial capacity, particularly given that over 70% received monthly pensions below 1400 RON. Awareness of partial reimbursements offered by the National Health Insurance House (CNAS) was limited, and those informed often expressed skepticism about the administrative complexity and actual accessibility of these benefits.

Logistical difficulties represented the second most common barrier to dental care. Many residents faced significant mobility impairments, relied on walking aids or wheelchairs, and lacked adequate transportation support from the institution. In numerous cases, the absence of a caregiver or escort made off-site dental appointments practically impossible, effectively isolating residents from external services. Furthermore, 52% of respondents (n = 36) noted that they lacked a caregiver or escort who could accompany them during dental visits, effectively rendering external appointments unfeasible.

Administrative and informational barriers were equally critical. Most participants were unaware of their eligibility for publicly funded dental services, while others described reimbursement procedures as excessively bureaucratic or inaccessible. Very few reported ever receiving information about oral health from care staff, nor did any facility involved in the study offer structured educational programs regarding dental hygiene or available public services.

Finally, psychological and emotional factors also contributed to the avoidance of dental care. Several residents reported anxiety and fear related to dental visits, often linked to previous negative experiences. Some perceived oral health problems as a natural part of aging, while others believed that treatment would be ineffective or unnecessary at their stage of life.

These cumulative findings indicate that the barriers faced by long-term care individuals are multifactorial and deeply interconnected. Economic hardship is compounded by physical frailty, while the absence of coordinated public initiatives exacerbates disparities in oral healthcare access. Without dedicated strategies to address these obstacles, elderly residents in long-term care remain functionally excluded from the national dental care system.

### 3.5. Perception of Oral Health and Quality of Life

The subjective impact of oral health on the daily lives of long-term care residents was a central focus of the present study. Residents were asked to evaluate how their oral condition affected essential functions such as chewing, speaking, social interaction, and overall well-being. Their responses revealed a profound and often overlooked burden that extended far beyond clinical findings.

More than two-thirds of participants, 67.5% (n = 47), reported experiencing difficulties in chewing or swallowing, largely due to edentulism or the use of ill-fitting dentures. Several residents described avoiding hard or fibrous foods, relying primarily on soft or mashed meals provided by the institution. This dietary limitation was frequently associated with poor nutritional intake and, in some cases, visible weight loss. Residents with multiple comorbidities, particularly those with diabetes or cardiovascular disease, expressed concerns that inadequate mastication was exacerbating their general health.

Aesthetic concerns were also prominent. Nearly half of the participants (48%; n = 34) reported that they avoided smiling, laughing, or speaking in public due to embarrassment about their dental appearance. Those with missing anterior teeth or fractured prostheses were especially affected. In informal interviews, several individuals recounted past experiences of social discomfort, including being reluctant to engage in group activities or refusing visits from family members due to self-consciousness.

Beyond functional and aesthetic challenges, the psychological impact of oral disease was notable. Thirty-nine percent (n = 27) of residents acknowledged that their oral discomfort contributed to social withdrawal, loss of confidence, or depressive symptoms. Some respondents reported feelings of being neglected or “forgotten” by the healthcare system, especially when comparing their access to services with the general population. While a minority of participants maintained good oral hygiene and showed resilience despite dental problems, the overall trend pointed toward a deterioration of oral health-related quality of life in most cases.

When asked to rate their overall oral health, 54.3% (n = 38) assessed it as “poor” or “very poor”, while only 8 residents considered their oral status satisfactory. The remainder (approximately 34%) offered neutral or mixed responses, often tied to low expectations or resignation regarding their aging and institutional context.

These findings confirm that, for many institutionalized elderly individuals, oral health is not a peripheral concern but a central determinant of dignity, nutrition, social interaction, and emotional well-being. Addressing their dental needs requires not only clinical intervention but also a shift in how oral health is prioritized within long-term care policy and daily institutional practice.

## 4. Discussion

### 4.1. Interpreting the Medical Needs

The clinical findings of our study confirm the presence of significant, yet largely unmet, oral health needs among institutionalized elderly individuals in Romania. The high prevalence of edentulism identified in our cohort underscores the chronic neglect of oral rehabilitation within institutional settings. This trend mirrors findings from Eastern European studies, which similarly attribute widespread tooth loss among institutionalized elders to limited access to prosthetic rehabilitation and preventive care [16]. This severe prosthetic under-coverage not only impairs masticatory function and aesthetics but is also strongly associated with malnutrition, social isolation, and decreased quality of life, factors consistently identified in the literature [17].

Although the rate of active caries observed in our sample may underestimate the true burden due to the visual-only assessment method, the trend aligns with international data identifying untreated root caries and periodontal problems as the dominant oral diseases in aging populations [18]. These conditions are frequently intensified by xerostomia, polypharmacy, and declining motor function, all of which are common among frail elderly individuals.

The occurrence of mucosal lesions, frequently linked to ill-fitting or worn dentures, further emphasizes the absence of systematic prosthetic maintenance and regular oral examinations. While most lesions were benign, their persistence increases discomfort and infection risk, particularly in residents with cognitive impairments or multiple comorbidities. Similar patterns have been documented in institutional settings across Europe, where inadequate screening and poor denture hygiene remain leading contributors to soft tissue trauma [19].

Overall, our findings support the broader understanding that the oral health status of long-term care individuals is not only a reflection of biological aging but also of systemic neglect. They reflect structural shortcomings (e.g., insufficient preventive protocols, lack of trained geriatric dental professionals), and the structural barriers within Romania’s public health infrastructure contribute to a silent, yet significant, burden of disease.

In summary, the oral health needs identified in this study go beyond isolated clinical issues. They represent a multidimensional health crisis, combining physical discomfort, reduced nutrition, aesthetic stigma, and diminished dignity, that requires integrated solutions at both clinical and policy levels.

### 4.2. Financial Inaccessibility of Basic Dental Treatments

In examining the financial inaccessibility of basic dental treatments for institutionalized elderly individuals in Romania, it becomes clear that both economic factors and systemic barriers significantly affect their access to dental care. A multifaceted understanding of these challenges is essential to developing effective interventions and policies that address the unique needs of this demographic.

One of the most significant barriers revealed by our study concerns the limited financial capacity of institutionalized elderly individuals to afford even the most basic dental services. Despite clear treatment needs, particularly for prosthetic rehabilitation and emergency interventions, most residents were unable to pursue care due to prohibitive costs. This pattern underscores a deeper structural inequity, where dental services remain largely privatized and unsupported by the public system.

Comparable challenges have been documented across Romania and throughout Central and Eastern Europe, where older adults face similar affordability constraints. As noted by Hage et al., dental care remains largely privatized in Romania, with limited public subsidies available for adult or geriatric populations [11]. Although the National Health Insurance House (CNAS) technically reimburses a portion of dental services, such as 60% of the cost for acrylic dentures, these benefits are insufficient in practice. Many elderly individuals are unaware of the reimbursement procedures or are discouraged by administrative hurdles and long wait times.

The absence of dedicated public funding for dental services in long-term care facilities further compounds existing inequities. In contrast to countries such as Germany and Sweden, where dental care for the elderly is integrated into comprehensive social health insurance systems, Romania continues to regard geriatric oral care as an individual financial responsibility [20,21]. As a result, oral health disparities deepen in institutionalized settings, where patients experience both higher need and lower access.

The financial strain on elderly individuals is exacerbated by limited income, often resulting in the prioritization of healthcare expenditures. As established in a study on barriers to dental care, many elderly individuals perceive dental treatment as a lower priority compared to other medical or household expenses, a mindset prompted by limited financial resources [22]. This prioritization arises from a necessity to allocate funds toward more immediate health concerns or essential living costs, leading to an acute level of unmet dental needs within this population [23]. Essentially, economic constraints significantly restrict the ability of elderly patients to seek out necessary dental care, leading to a downward spiral of oral health deterioration and increasing healthcare complications.

Evidence also indicates that financial support for elderly individuals often depends on family or community resources, particularly in contexts where pension systems are inadequate or inconsistent [24]. For many institutionalized residents, this reliance creates an additional barrier to seeking dental care, as treatment costs are perceived as a financial burden on relatives. Within long-term care facilities, this dynamic is amplified by limited mobility and restricted access to external providers [25]. The intersection of physical frailty and financial dependency thus forms a critical barrier to oral healthcare, reinforcing social and health inequities that extend far beyond the institutional setting.

The economic barriers to dental care extend beyond individual financial hardship and reflect deeper systemic shortcomings within the national healthcare framework. In Romania, as in many other regions, oral health services remain poorly integrated into public healthcare, and substantial out-of-pocket expenses persist even for procedures nominally covered by insurance [26]. This disconnect between formal service availability and real accessibility underscores a structural inequity: dental care exists in principle but remains unattainable in practice for many older adults. As highlighted in previous studies, affordability concerns often outweigh perceived need, leading elderly individuals to forgo treatment even for urgent conditions [23].

A pertinent solution involves exploring outreach programs that could bridge the accessibility gap. Implementing community-based dental outreach initiatives could serve to reduce disparities in access and increase the financial feasibility of obtaining care for the elderly. Such approaches have been shown to bolster service utilization among underserved populations, partly by mitigating transportation and geographical barriers, and partly by providing cost-effective care [27,28]. Additionally, legislative changes aimed at increasing insurance coverage and financial assistance for dental care could alleviate some of these barriers, allowing elderly patients to prioritize oral health without sacrificing their financial stability [22,29].

Moreover, the integration of telehealth and teledentistry into the care model for the elderly could also play a significant role in alleviating financial burdens. By moving some forms of dental consultations online, institutions could reduce costs for both providers and patients, increasing access to care without the extensive resources typically required for in-person visits. Such initiatives promote a model of care that emphasizes accessibility while also addressing systemic inequities in healthcare provision for the elderly [30].

These findings challenge the notion that limited reimbursement schemes can ensure equity in oral healthcare. For medically dependent and economically vulnerable older adults, financial cost represents not merely a barrier but a form of exclusion. Without targeted policy reform and sustainable public financing, the right to oral health for institutionalized elderly individuals will remain a legal formality rather than a lived reality.

In summary, the convergence of economic hardship, structural barriers, and insufficient legislation creates a systemic environment that perpetuates oral health inequities among Romania’s aging population. Addressing these challenges requires coordinated efforts across governmental, professional, and community sectors to expand financial accessibility, promote preventive services, and integrate oral health into broader geriatric care strategies.

### 4.3. Structural Deficits in Oral Health Delivery

Beyond economic limitations, the structural barriers to delivering dental care in institutional settings are a major contributing factor to oral health disparities among the elderly. Our findings indicated that none of the residential care units included in the study had on-site dental infrastructure or permanent collaborations with dental service providers. Moreover, only one of the facilities had received a visit from a mobile dental unit in the past year—a service that was reported as irregular and unsystematic.

This absence of integrated dental services within care institutions reflects a broader systemic deficiency in Romania’s public health architecture. According to Alexa et al., even in urban areas, dental care provision in elderly facilities is fragmented, and there is no national protocol for the inclusion of dental services in geriatric institutional settings [31]. The issue is further exacerbated by the lack of specialized training among care staff and general practitioners in recognizing and managing oral health problems in older adults.

At the European level, the structural integration of dental services into long-term residential care varies considerably. In countries such as Denmark, the Netherlands, and Sweden, multidisciplinary models embed dental professionals within geriatric care teams, ensuring regular on-site assessments and continuity of care [20,21,32]. By contrast, Romania lacks a coordinated framework for such integration. Existing mobile dental initiatives remain sporadic and unsustainable, relying largely on the efforts of non-governmental organizations or short-term local projects. This fragmented approach limits continuity, prevents systematic prevention, and perpetuates inequality in access to essential oral health services. The lack of oral health screening programs and preventive strategies within institutional protocols is also evident. In our study, none of the participants had undergone a routine dental check-up in the last 12 months unless prompted by acute symptoms. The absence of standardized preventive practices (e.g., oral hygiene support, plaque control programs, prosthetic maintenance) significantly increases the burden of disease and leads to irreversible conditions that could otherwise be avoided.

Moreover, the infrastructure needed to accommodate dental visits, even external ones, is frequently missing. Many institutions lack proper wheelchair access, transportation assistance, or coordination protocols to ensure continuity of care. These deficits create a situation where elderly individuals, particularly those with cognitive or physical impairments, become completely dependent on external resources for dental attention, resources which, in practice, are almost entirely unavailable.

Overall, the Romanian institutional care system for the elderly lacks the organizational, professional, and logistical components necessary to ensure basic oral health rights. The development of mobile units, mandatory annual oral check-ups, and professional training for caregiving staff must become national priorities if structural inequality is to be meaningfully addressed.

### 4.4. Legislative Gaps and Under-Implementation in Romania

The legislative context governing oral healthcare for institutionalized elderly individuals in Romania reveals persistent gaps and weak implementation, which substantially contribute to their unmet needs. Existing laws address general health and social assistance but fail to recognize oral health as a distinct component of geriatric care. This omission limits both policy coherence and service delivery, leaving dental health largely unregulated within long-term care settings.

Although Law no. 17/2000 ensures access to medical, social, and psychological services for older adults in residential institutions [33], it does not explicitly include oral healthcare or define mechanisms for its provision. As a result, oral health remains outside the operational scope of institutional care, reflecting a broader pattern of systemic neglect rather than a lack of legal intention.

Although Romanian legislation guarantees access to healthcare for institutionalized elderly individuals, it lacks concrete mechanisms for implementation. Law no. 17/2000 does not provide operational protocols or dedicated funding for dental interventions, and Article 20 omits oral care from the mandatory services that residential institutions must deliver [33]. This absence of enforceable provisions translates directly into the systemic neglect observed in practice—our findings confirmed that none of the residents received preventive dental assessments or regular oral hygiene support.

By contrast, several European countries have embedded oral healthcare within their geriatric care frameworks. In Sweden, national guidelines require dental assessments as part of every residential care plan, with public insurance covering much of the cost for prosthetic rehabilitation [34]. Similarly, Germany’s statutory health insurance integrates routine dental visits into long-term care programs and financially incentivizes institutions to maintain partnerships with mobile dental units [35]. These examples highlight Romania’s regulatory gap and the need for policy models that link legal provisions to enforceable practice. Although Romania’s National Health Insurance House (CNAS) does offer limited reimbursements for certain dental procedures, access is often obstructed by bureaucratic complexity, insufficient contract coverage, and the absence of in-house services in care homes.

The under-implementation of existing legal frameworks is also evident in the lack of monitoring or sanctions. While Law 17/2000 allows for inspection of residential facilities, there is no specific provision for assessing compliance with oral health standards, nor are there incentives for institutions to provide routine dental support [33]. This regulatory void leads to a situation in which the oral health rights of elderly residents exist in principle, but not in practice.

To address this discrepancy, Romania must move beyond generic legal formulations and adopt specific oral health provisions within its geriatric care legislation. These should include:

○ Mandatory annual oral health assessments for institutionalized individuals.
○ Integration of dental services in the list of essential health services under CNAS.
○ Development of mobile dental units subsidized by the state.
○ Professional training in geriatric dentistry for institutional caregivers.

Until such measures are formally adopted and enforced, the systemic exclusion of oral healthcare from the Romanian elderly care model will persist, with clear consequences for the dignity, nutrition, and health of some of the country’s most vulnerable citizens.

A major shortcoming of the current Romanian legal framework is the absence of explicit provisions addressing geriatric oral healthcare in institutional settings. Although health legislation is broad in scope, it fails to define specific standards for oral hygiene maintenance among long-term care residents. As a result, caregivers, despite generally adequate medical training, often lack the knowledge or guidance needed to manage the oral health needs of elderly individuals. Prior studies confirm that systematic education on oral hygiene remains insufficient within caregiving curricula, reinforcing the gap between legislative intent and actual implementation in care facilities [36,37].

Economic constraints represent a central obstacle to implementing effective oral health initiatives in Romania’s long-term care system. Many residents experience declining functional capacity and autonomy, while institutions themselves face chronic underfunding that prevents prioritization of dental services [38,39]. This reflects a broader structural weakness within the national healthcare framework, where limited budgets and low awareness of oral health needs contribute to persistent neglect [40]. Addressing these deficiencies requires a reassessment of funding priorities and a commitment to allocating dedicated resources for geriatric oral care.

Equally problematic is the inconsistency between policy and practice. Although Romanian legislation sets general health standards for residential institutions, compliance and oversight remain weak. Numerous facilities lack formal partnerships with dental providers, resulting in fragmented and largely reactive care [41]. Without mechanisms to monitor enforcement or ensure accountability, preventive dental services are rarely implemented, leaving residents vulnerable to conditions that could otherwise be managed through early intervention [42].

Moreover, the training and educational gaps among health care providers further contribute to the landscape of under-implementation. The existing curricula for nursing and caregiving staff often do not encompass comprehensive topics related to oral health, which is critical for the elderly population [37]. The lack of training and comfort in performing oral care tasks among nursing home staff not only exacerbates neglect but also perpetuates negative attitudes towards the importance of oral hygiene [40]. Addressing this deficiency requires legislative action aimed at integrating oral health education into caregiver training programs effectively, ensuring that staff possess the requisite knowledge and skills to provide adequate care.

The cumulative effects of legislative gaps and weak implementation are reflected in the poor oral health outcomes observed among long-term care residents in Romania. Studies consistently show that a large proportion of institutionalized elderly individuals suffer from untreated oral conditions that contribute to systemic complications, including malnutrition and chronic disease, ultimately diminishing their quality of life [36,43,44]. These findings underline the urgent need for legislative reform that moves beyond declarative rights to enforceable standards of care, integrating preventive measures and sustainable service delivery.

As Romania’s population continues to age, the consequences of these systemic shortcomings will intensify unless decisive action is taken. Developing policies that address the specific needs of institutionalized elders requires active collaboration among healthcare professionals, policymakers, and community stakeholders. Interdisciplinary cooperation is essential to designing interventions that are both effective and sustainable, ensuring that oral health becomes a core component of comprehensive geriatric care [45].

Overall, the examination of legislative gaps and under-implementation in Romania concerning oral health care for long-term care residents reveals a multifaceted challenge characterized by inadequate policy frameworks, economic barriers, poor compliance, and insufficient caregiver education. Addressing these issues requires comprehensive reforms that align legislation with the actual needs of elderly care. This should encompass improved funding for health services, enhanced caregiver training focused on oral health, and regular audits of institutional compliance with health standards. Only through a concerted effort involving policymakers, caregivers, and healthcare systems can we hope to secure better health outcomes for Romania’s aging population and ensure that their rights to health and dignity are upheld effectively.

### 4.5. Comparative European Perspectives and Best Practices

The issue of oral health care for the institutionalized elderly population in Romania necessitates examining comparative perspectives and best practices across Europe. Various countries have developed distinct legislative frameworks, health policies, and innovative practices aimed at improving oral health outcomes for elderly individuals residing in care facilities.

In Sweden, dental care for elderly populations is partially subsidized, with county councils offering financial assistance that reduces out-of-pocket costs for those requiring extensive care. However, studies show that only a fraction of eligible individuals use these benefits due to administrative complexity and limited awareness [46]. This illustrates a broader European challenge: although many countries have established supportive legal frameworks, implementation barriers continue to restrict access. Romania could draw from this experience by coupling legislative reforms with public awareness campaigns and simplified administrative procedures to ensure that existing benefits are both accessible and effectively utilized.

Germany has implemented a structured approach to preventive oral health care aimed at the elderly through legislative initiatives that outline the responsibilities of caregivers and institutions. Specific laws mandate regular dental examinations as part of broader health assessments for elderly individuals in care settings [47]. This preventive care model significantly reduces the rate of dental diseases and improves overall health outcomes, echoing findings from previous studies that link poor oral health with a decline in both physical health and quality of life [17,43]. By adopting a similar preventive framework that integrates oral assessments into comprehensive health screenings, Romania could address the critical health needs of its elderly population more effectively.

The Netherlands provides a strong example of innovation in geriatric oral healthcare through its comprehensive training programs for caregivers in long-term care facilities. These initiatives focus on improving knowledge of oral hygiene practices and promoting awareness of the role of oral health in overall well-being [48]. Evidence indicates that such educational interventions not only strengthen caregiver competence but also lead to measurable improvements in residents’ oral health outcomes [49]. Integrating similar mandatory training within Romanian legislation could enhance the quality of institutional care and ensure more consistent oral hygiene support for elderly residents. In addition to educational programs, the Netherlands has also integrated teledentistry into their care model, offering remote consultations and assessments, which have been shown to be just as effective as traditional in-person visits for diagnosing dental conditions [30]. This approach is especially beneficial for institutionalized elderly individuals who may face mobility challenges and other barriers to accessing conventional dental care. Implementing teledentistry could expand access to necessary oral health services in Romania, addressing geographic disparities in healthcare access for the elderly [46].

South Korea offers a noteworthy example of integrating oral healthcare for seniors within its National Health Insurance (NHI) framework, which explicitly includes denture coverage for older adults. This policy directly addresses the functional and nutritional challenges associated with edentulism, improving both comfort and quality of life [29]. Evidence shows that expanding insurance benefits significantly reduces unmet dental needs and increases care utilization among the elderly [50]. Adopting similar reimbursement mechanisms in Romania could enhance the affordability of essential dental services and help bridge existing gaps in access to geriatric oral healthcare.

Moreover, an essential aspect of successful implementations in both South Korea and other European countries involves the empowerment of caregivers through training and resources. Proper education for caregivers has proven to have direct effects on enhancing the oral hygiene practices of elderly individuals, mitigating the risks associated with poor oral health [22]. Hence, a concerted effort in Romania to invest in caregiver education and develop a robust support network for health professionals in institutional settings would likely yield significant improvements in elderly dental health.

Across Europe, oral health is increasingly recognized as an integral component of general health policy. The European College of Gerodontology recommends incorporating oral assessments into routine medical evaluations and extending public insurance coverage to include essential dental services for institutionalized older adults [47]. Aligning Romania’s health policies with these standards would promote a more cohesive and equitable approach to geriatric care.

Globally, financial barriers remain a primary deterrent to seeking dental treatment among the elderly [23]. Many countries have mitigated this challenge through cost-reduction policies and targeted subsidy programs, proving the effectiveness of financial support in improving access. Romania could adopt similar strategies—combining Sweden’s subsidized care model with South Korea’s expanded insurance coverage—to reduce economic disparities and enhance oral health outcomes within its aging population.

The legislative frameworks across Europe also highlight the need for collaborative efforts between the health and social care sectors. For instance, integrated health care models that include both medical and social components have been shown to enhance oral health care delivery by improving coordination between different care providers [51]. In Romania, the establishment of integrated care pathways that encompass both oral and general health for the elderly could further facilitate access to necessary services and resources.

Lastly, while examining barriers to effective oral healthcare, studies have illuminated various systemic and organizational challenges that can impede the delivery of care [52]. Understanding and addressing these barriers through policy reforms, such as increasing the training and readiness of dental care providers in geriatric dentistry, is crucial for enhancing care that aligns with the needs of an aging population, specifically within institutionalized settings [53].

The comparative European perspectives on oral health care for the elderly reveal significant insights that can inform Romania’s future direction in this domain. The legislative frameworks, preventive care strategies, caregiver training programs, and innovative service delivery models provide a solid foundation upon which Romania can build to enhance its oral health care services for institutionalized elderly individuals. By adopting best practices from other European nations and tailoring them to its specific context, Romania has the potential to substantially improve the oral health status and quality of life for its aged population.

### 4.6. Impact on Quality of Life

Oral health plays a critical role in the well-being of elderly individuals, influencing not only their ability to eat and speak but also their dignity, self-image, and social participation. Our study confirmed that a large proportion of them experience a considerable decline in their oral health-related quality of life (OHRQoL), with direct consequences on their physical and emotional states. The OHRQoL among long-term care individuals significantly influences their overall quality of life, impacting both psychological well-being and social interaction.

More than two-thirds of participants reported significant difficulties in chewing or swallowing, limiting their food choices, and contributing to poor nutritional intake. This finding is consistent with previous research indicating that edentulism and ill-fitting prostheses can lead to malnutrition, unintended weight loss, and exacerbation of systemic conditions such as diabetes and cardiovascular disease [54].

Furthermore, nearly half of the residents expressed discomfort with their appearance due to missing teeth or damaged dentures, leading them to avoid smiling or speaking in social settings. This loss of confidence was frequently accompanied by social withdrawal, especially during communal meals, visits from family members, or group activities. Studies have shown that such psychosocial impacts are particularly profound in institutional environments, where opportunities for social interaction are already limited [55].

The psychological burden of poor oral health was also evident in the residents’ perception of neglect. Many participants reported feeling forgotten by the health system or abandoned, sentiments often linked to the invisibility of dental care within general medical services.

Beyond individual experiences, the decline in oral function contributes to a loss of autonomy, a core component of quality of life in older adults. When an elderly person loses the ability to chew, speak clearly, or control oral discomfort, their capacity for self-care and participation in daily life diminishes.

Multiple studies emphasize the strong link between oral health and overall well-being, showing that poor oral hygiene can contribute to psychological distress, social withdrawal, and diminished self-esteem. Among institutionalized elderly individuals—who often face declining physical health and mobility—maintaining oral health is essential for preserving dignity, autonomy, and social participation [36,56,57].

Unmet dental care needs remain widespread in residential settings, where the oral health status of residents is frequently inadequate. Chiesi et al. highlighted the importance of systematic screenings and caregiver education programs to ensure consistent and effective oral hygiene support [56]. In the absence of such preventive measures, oral conditions tend to worsen, leading to functional limitations (e.g., impaired chewing, speech) that directly compromise nutrition, systemic health, and overall quality of life [58,59].

The emotional ramifications of poor oral conditions among institutionalized elderly cannot be overlooked, as evidenced by studies that illustrate the correlation between oral health status and self-perceived quality of life. An incisive link between xerostomia (dry mouth), denture-related problems, and social interactions has been documented, where individuals suffering from these issues report lower life satisfaction scores [57,60,61]. Moreover, the utilization of removable dentures and their proper management can directly enhance the quality of life, allowing for improved eating capabilities and reduced discomfort [62,63].

The complexities of aging, coupled with potential socioeconomic barriers, further compound the challenges of maintaining oral health. Elderly individuals often contend with low income, lack of education, and diminished access to health services—factors that collectively reduce their ability to receive necessary dental interventions. This access issue is particularly pronounced in low and middle-income countries, where the systemic neglect of oral health services for the elderly can lead to significant declines in OHRQoL [59,64]. Research indicates that financial constraints and systemic healthcare failures result in inadequate treatment, leading to deterioration in both oral and overall health [48,59,61].

National policies and legislative frameworks aimed at improving healthcare delivery systems play a critical role in enhancing the quality of life for institutionalized elderly populations. Existing models suggest that integrating a community-centered approach, where dental health professionals work closely with caregivers and institutional staff, can foster a holistic framework to address the multitude of oral health needs of the elderly [59,65]. Additionally, funding for mobile dental services and education of caregivers on oral hygiene practices could facilitate regular and thorough oral care, ultimately leading to improved health outcomes [59,65].

In Romania, establishing a robust legislative framework for quality dental care must go hand in hand with continuous training for caregiving staff. Evidence shows that tailored oral health education programs in long-term care settings markedly improve the quality and consistency of care when caregivers possess adequate knowledge and skills [48,56]. This underscores the need for policies that extend beyond service accessibility to include systematic professional development and institutional accountability.

Importantly, the concept of oral health-related quality of life (OHRQoL) transcends clinical metrics and reflects broader ethical obligations toward ensuring dignity and well-being in aging populations [30]. Implementing systems that assess and enhance OHRQoL would enable a more holistic understanding of the psychosocial dimensions of oral health, guiding interventions that simultaneously promote physical health, emotional resilience, and social inclusion among institutionalized elders [58,66].

Improving oral healthcare for institutionalized elderly individuals in Romania requires an interventional approach that goes beyond conventional treatment models. Strengthening caregiver education, expanding service accessibility, and embedding oral health within national health and social policies are essential steps toward sustainable improvement. Such measures can meaningfully enhance oral health–related quality of life (OHRQoL) and support a more dignified aging process.

Ultimately, oral health among institutionalized elders must be recognized not solely as a clinical concern, but as a determinant of equity, dignity, and human rights. Addressing it demands a holistic perspective that integrates physical, emotional, and social dimensions of well-being ensuring that care for the elderly upholds both their health and their humanity.

## 5. Conclusions and Future Perspectives

This study provides a comprehensive overview of the oral health status, treatment needs, and systemic barriers faced by institutionalized elderly individuals in Timisoara, Romania. The findings reveal a deeply rooted disparity between medical necessity and service accessibility, highlighting the extent to which oral healthcare remains a neglected dimension of geriatric care in long-term residential facilities.

The clinical data indicate a high prevalence of total edentulism, untreated caries, and the absence of functional prosthetic rehabilitation. These oral conditions significantly impair nutrition, communication, and psychosocial well-being, contributing to a measurable decline in quality of life. Beyond physical discomfort, many residents expressed emotional distress and social withdrawal, driven by shame, aesthetic concerns, or a perceived loss of dignity, underscoring the profound impact of oral health on self-perception and human interaction.

Equally concerning is the low rate of dental service utilization. Financial limitations, logistical constraints, and bureaucratic opacity converge to effectively exclude institutionalized older adults from receiving even basic dental care. Although Romanian legislation (notably Law no. 17/2000) guarantees access to healthcare in residential facilities, the absence of specific provisions for oral health results in inconsistent or non-existent implementation. Without regulatory obligation or designated funding, oral healthcare remains marginalized.

In comparison with several European models (such as those in Sweden, Germany, or the Netherlands), Romania falls short in integrating oral care within the broader health framework for the elderly. These countries demonstrate that coordinated, multidisciplinary, and publicly funded approaches are both feasible and cost-effective, with proven outcomes in terms of reduced systemic complications, improved patient satisfaction, and ethical care standards. Based on these findings, several future directions emerge:

(i) Legislative reform is essential. Romania must revise and expand its legal framework to include explicit recognition of oral health as a component of institutional medical care, with enforceable standards and accountability mechanisms.
(ii) Structural integration of dental services within residential care settings is urgently needed. This may include the development of state-funded mobile dental units, tele-dentistry support for remote assessments, and periodic on-site dental evaluations.
(iii) Financial accessibility must be enhanced through simplified reimbursement procedures, increased service coverage under CNAS, and the creation of special public subsidies for prosthetic rehabilitation for low-income elderly individuals.
(iv) Training and awareness programs for institutional caregivers and healthcare staff should be developed to improve daily oral hygiene practices, early detection of lesions, and referral mechanisms.
(v) Research and monitoring should be continued and expanded. National surveys and clinical audits can provide updated data to inform public health planning and measure the impact of future interventions.

Ultimately, ensuring oral health for institutionalized elderly individuals is not solely a clinical responsibility but a reflection of societal priorities. The right to eat comfortably, speak without shame, and smile with dignity must be considered fundamental, not optional, for every person, regardless of age or income. Oral healthcare should no longer be the forgotten element of geriatric policy in Romania.

## Figures and Tables

**Table 1:** Inclusion and exclusion criteria for the elderly patients from the residential care center.

Criteria	Included	Excluded
Age ≥ 65 years	✓	
Permanent resident in the care home	✓	
Ability to provide informed consent or presence of a legal guardian	✓	
Medically stable and available for dental examination.	✓	
Terminal illness		✓
Recent hospitalization (<2 weeks before evaluation)		✓

**Table 2:** The most prevalent comorbidities in elderly patients from the residential care center.

Comorbidity	Number of Patients (%)
Cardiovascular diseases	42 (60%)
Type 2 *Diabetes Mellitus*	21 (30%)
Osteoarticular disorders	19 (27.1%)
Cognitive decline or dementia	15 (21.4%)
Respiratory diseases	11 (15.7%)
Gastrointestinal diseases	9 (12.9%)
Depression or mental illness	8 (11.4%)
Malnutrition	6 (8.6%)

**Table 3:** Oral health conditions among institutionalized elderly residents (n = 70).

Parameter	n	%
Total edentulism	47	66.6
Partial edentulism	15	21.4
Functional natural dentition	8	12
Completely edentulous without dentures	30	42.9
Damaged/poorly fitting dentures	20	28.6
Functional complete dentures	20	28.6
Active dental caries	27	38.6
Periodontal disease	31	44.7
Oral mucosal lesions	13	18.2

**Table 4:** Reported barriers to accessing dental care among institutionalized elderly residents (n = 70).

Category	Description	n	%
Economic	Unable to afford dental treatments Limited awareness of CNAS reimbursement Low pension income	59	84
Logistical	Transportation difficulties Physical mobility limitations Lack of escort to appointments	42	60
Administrative	Unaware of entitlements Bureaucratic complexity Absence of oral health education	54	77
Psychological	Fear Anxiety Resignation toward dental care	15	21

## Data Availability

The original contributions presented in the study are included, further inquiries can be directed to the corresponding author.
